# A New Pharmacophore Model for the Design of Sigma-1 Ligands Validated on a Large Experimental Dataset

**DOI:** 10.3389/fphar.2019.00519

**Published:** 2019-05-31

**Authors:** Rosalia Pascual, Carmen Almansa, Carlos Plata-Salamán, José Miguel Vela

**Affiliations:** ESTEVE Pharmaceuticals S.A., Drug Discovery and Preclinical Development, Barcelona, Spain

**Keywords:** sigma-1, crystal structure, 5HK1, pharmacophore model, docking, virtual screening

## Abstract

The recent publication of the σ1R crystal structure is an important cornerstone for the derivation of more accurate activity prediction models. We report here a comparative study involving a set of more than 25,000 structures from our internal database that had been screened for σ1R affinity. Using the recently published crystal structure, 5HK1, two new pharmacophore models were generated. The first one, **5HK1–Ph.A,** was obtained by an algorithm that identifies the most important receptor-ligand interactions including volume restrictions enforced by the atomic structure of the recognition site. The second, **5HK1–Ph.B,** resulted from a manual edition of the first one by the fusion of two hydrophobic (HYD) features. Finally, we also docked the database using a high throughput docking technique and scored the resulting poses with seven different scoring functions. Statistical performance measures were obtained for the two models, comparing them with previously published σ1R pharmacophores (Hit Rate, sensitivity, specificity, and Receiver Operator Characteristic) and **5HK1–Ph.B** emerged as the best one in discriminating between active and inactive compounds, with a ROC-AUC value above 0.8 and enrichment values above 3 at different fractions of screened samples. **5HK1–Ph.B** also showed better results than the direct docking, which may be due to the rigidity of the crystal structure in the docking process (i.e., feature tolerances in the pharmacophore model). Additionally, the impact of the HYD interactions and the penalty for desolvating ligands with polar atoms may be not adequately captured by scoring functions, whereas HYD groups filling up such regions of the binding site are entailed in the pharmacophore model. Altogether, using annotated data from a large and diverse compound collection together with crystal structure information provides a sound basis for the generation and validation of predictive models to design new molecules.

## Introduction

The sigma-1 receptor (σ1R) is an intracellular chaperone protein, expressed in CNS regions and known to regulate Ca^2+^ signaling and cell survival. The σ1R gene encodes a 24 kDa protein of 223 amino acids anchored to the endoplasmic reticulum (ER) and plasma membranes (Maurice and Su, 2009). The σ1R sequence has no homology with other mammalian proteins and is structurally and functionally different from other target classes. The σ1R is also unique in that it exerts molecular chaperone activity and interacts with diverse proteins to modulate their functions. Accordingly, the σ1R is involved in many physiological functions, including inter-organelle signaling (Su et al., 2010). Its activity can be regulated by ligands in an agonist/antagonist manner (Hayashi et al., 2011). Just as examples, the σ1R modulates opioid analgesia through physical protein-protein interactions, with σ1R antagonists enhancing and σ1R agonists inhibiting the antinociceptive effect of opioids, and σ1R antagonists reproduce the pain-protective phenotype of σ1R knockout mice when administered to wild-type mice (Zamanillo et al., 2013).

**FIGURE 1:**
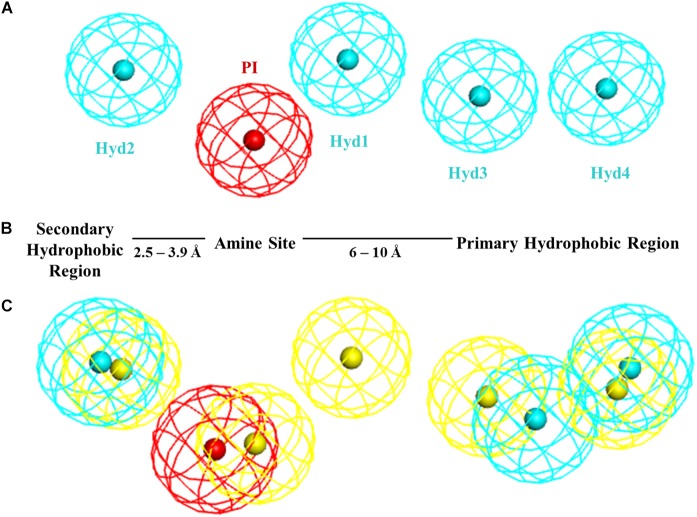
**(A)** Langer–Ph. **(B)** Glennon–Ph; **(C)** Comparison of **5HK1–Ph.A** (without exclusion spheres) with Langer–Ph (yellow).

Until its recent crystallization, little was known about the σ1R 3-dimensional (3D) structure and the rational design of σ1R modulators mostly relied on ligand-based approaches. Based on a series of diphenylalkylamines, a first 2D-pharmacophore model (Glennon–Ph) for the σ1R was designed in the early 90’s (Glennon et al., 1994) consisting in a positive ionizable (PI) group (i.e., a basic amino group) and two opposite hydrophobic (HYD) regions at 2.5–3.9 Å and 6–10 Å without any angle constrain (Figure 1). This qualitative model has been very useful as a guide to medicinal chemists for the design of new ligands. In 2004, a Sybyl 3D-pharmacophore model (Gund–Ph) was derived based on the alignment of PD144418, spipethiane, haloperidol and (+)-pentazocine (Gund et al., 2004). It consists in an aromatic region and a nitrogen atom that acts as hydrogen bond acceptor, as primary requirement for binding, and a polar feature representing an oxygen or sulfur atom as secondary binding interaction. In 2005, Langer’s group developed a 3D-pharmacophore model (Langer–Ph) based upon 23 structurally diverse molecules with σ1R K_i_ values between 10 pM and 100 μM (Laggner et al., 2005). The model was generated with the HypoGen algorithm of Catalyst (Catalyst 4.9, 2003) and it consists in one PI and four HYD features (Figure 1). The model is in good agreement with Glennon’s one but lacks the secondary polar binding region of Gund–Ph. Another HypoGen derived model (Zampieri–Ph) was published in 2009 using a series of 31 benzo[d]oxazol-2(3H)-one derivatives (Zampieri et al., 2009). The model contains one hydrogen bond acceptor (HBA), two hydrophobic aromatic features (HYD-AR), one HYD feature and one PI group. It is also in agreement with Glenon–Ph concerning distances among the PI feature and any HYD group, but it includes an additional polar/hydrogen bond acceptor feature as hypothesized by Gund. Langer–Ph and Zampieri–Ph share feature type and number (except for the additional HBA and the differentiation of one HYD to aliphatic HYD). Reported distances from the PI group to HYD features are similar, but not so much as it regards to their disposition and angles. Using the MOE Pharmacophore Elucidation routine, Wünsch’s group aligned a training set of 66 spirocyclic derivatives to generate an additional pharmacophore model (Oberdorf–Ph) with four annotation points: aromatic, HYD, PI and HBA (Oberdorf et al., 2010). In 2012, another σ1R 5-features model for a series of 32 N-substituted azahexacyclododecanols was developed using the Phase program provided in Maestro (Banister et al., 2012). Its composition of HYD, PI and HBA features is in accordance with previous published models, but again with particular pairwise distances and angles. In summary, all the available pharmacophoric models share the presence of a PI and several HYD features with variations in distances and angles, and all of them, except Glenon’s and Langer’s ones include the presence of a polar group.

The first σ1R homology model was published in 2011 by Pricl’s group (Laurini et al., 2011). It was built taking as reference non-overlapping segments of four crystalized proteins with ≥30% sequence identities to the σ1R. The N-terminal domain (residues 1–16) was built *de novo* and the four fragments were joined, generating and ranking alternative models for the loop portions in each junction zone. This initial 3D model was then subjected to refinement by molecular dynamics and a putative binding site was identified. The refined σ1R homology model was then used for docking and binding affinity determination of a series of bioactive ligands and reference σ1R ligands via the MM/PBSA methodology, as well as for the design of new ligands and their ranking for receptor affinity (Laurini et al., 2012, 2013). Later on, another σ1R homology model was published, with results based on only the cold-active aminopeptidase, (PDB code 3CIA), also used by Pricl’s group among template structures, wherein two distinct but closely proximal binding sites were suggested from docking studies of pentacycloundecylamines using MOE (Geldenhuys et al., 2013).

In 2016, the first crystal structure of the human σ1R was published in complex with two ligands, PD144418 and 4-IBP (Schmidt et al., 2016). More recently the same group reported co-crystallization with additional compounds (Schmidt et al., 2018). The crystal structure shows an overall trimeric receptor arrangement, with a single transmembrane helix in each protomer, and each protomer binding a single ligand molecule. The single-pass transmembrane architecture was surprising in view of the widely accepted two-pass transmembrane architecture, compatible with or suggested from fluorescent tags and immunocytochemistry (Aydar et al., 2002; Hayashi and Su, 2007), radioiodinated photoprobe (Fontanilla et al., 2008) or solution circular dichroism-nuclear magnetic resonance (Ortega-Roldan et al., 2015) studies, although a single transmembrane segment close to the N-terminus and coded by exon 2 had already been suggested from the very beginning by hydropathy analysis of the amino acid sequence (Hanner et al., 1996; Kekuda et al., 1996; Seth et al., 1997; Prasad et al., 1998).

Taking advantage of the information and resolution provided by the X-ray crystallographic structure, we explored its contribution to the prediction of binding affinities in virtual screening conditions compared to previous pharmacophore models. To this aim we developed two new σ1R pharmacophore models using the structural information revealed by the crystal structure, which was also used for docking studies in several conditions. Additionally we reproduced most of the published σ1R pharmacophore models and compared their performance in front of a fraction of our chemical database, experimentally assayed for σ1R affinity, containing more than 25,000 unique structures. To the best of our knowledge this is the first time that such a large compound dataset is used for establishing the predictive value of σ1R models.

## Materials and Methods

### Protein Preparation

The recently crystalized σ1R structure (PDB = 5HK1) was prepared using Discovery Studio 16 (Dassault Systèmes BIOVIA, 2016a). Sulfate ions and oleoyl-glycerol molecules were removed, as well as all waters, since no key water molecules were observed within the binding site. Incomplete side chains were added, the structure was typed with the CHARMm forcefield and atoms were ionized according to the predicted p*K* at pH = 7.4, using the ‘calculate protein ionization and residue p*K’* protocol. The charge of Asp126 was set to zero, allowing a hydrogen bond with the charged Glu172 as previously hypothesized (Schmidt et al., 2016). Subunit B of the trimeric structure was selected for further calculation as it shows the lowest average isotropic displacement. However, very similar results should be obtained using any of the other two subunits, as the RMSD of the 3 subunits superimposed by C-alpha pairs of residues within 5 Å distance to the ligand has a value of 0.25 between chains A and B and of 0.18 between chains B and C (RMSD superimposed using the whole chains is a bit higher due to the different bending of the helices).

### Ligand Databases Collection and Preparation

All in-house characterized compounds for σ1R binding together with their data were retrieved from ESTEVE’s internal Activity Base database (IDBS, 2016). This made up a total of 25,676 unique structures. Compounds were obtained in the neutral form, as salts had been already striped in the registration process. Then a 3D multiconformational database was built with Catalyst as implemented in Discovery Studio 2016, using the BEST methodology (Smellie et al., 1995; Kirchmair et al., 2006). 3D conformational generation was launched from Pipeline Pilot 2016 (Dassault Systèmes BIOVIA, 2016b). Special attention was given to correctly retain the stereochemical information of the compounds. Both chirality options were included in the conformation generation process for racemic mixtures. In the case of enantiomeric mixtures with grouped stereocenters, Catalyst is not able to take the stereochemistry-related information into account for conformer generation. Hence, different 3D entries for each of those compounds with the stereochemical combination defined by the stereo-groups were created, generating conformations specifically for each of them and joining them afterward with the same compound identifier. Compounds with fused cyclopropyl groups as well as some substituted cyclobutyl derivatives cannot be treated by the BEST algorithm. In this case conformations were built by systematic search using a default torsion increment of 60 for sp3-sp3 and sp3-sp2 bonds and of 180 for sp2-sp2, followed by minimization using the MMFF force field. The final database generated, consisting of 3,707,672 conformers, was used as input for pharmacophoric screening.

Additionally a second multiconformational database of the same compounds, but ionized, was built. To do so, basic pKa constants were calculated for all compounds using both ACD-classic and ACD-Galas (ACD/Labs, 2014). A Pipeline Pilot protocol was designed to generate a pool of different ionization states for each compound, by protonating basic points with pKa values above 5 or unprotonating acidic points below 5 successively on the previous state, and adding the resulting ionized structure to the pool. The protocol was run for the ACD-classic and ACD-Galas generated values, and both output structures were merged and duplicate ionization states removed. Finally, the same procedure described above was followed, obtaining a new database with 7,573,004 conformers.

For the purpose of this work, structures were classified as actives when their K_i_ value was equal or under 1 μM (18.6% of the samples; 4766 structures) or as inactives in the contrary cases or when K_i_ values had not been determined because their percentage inhibition at 1 μM was under 50% (81.4% of the samples; 20,910 structures).

### Pharmacophore Generation

The receptor-ligand pharmacophore generator job implemented in Discovery Studio 16 was run on the prepared subunit B of the σ1R with the co-crystallized ligand PD144418 to obtain **5HK1–Ph.A**. The algorithm (Sutter et al., 2011) generates pharmacophore models from the features that correspond to the receptor-ligand interactions, identifying in a first step all ligand features and pruning then those features that do not match the protein-ligand interactions. It additionally places as well excluded volumes to represent the steric aspect of the protein. **5HK1–Ph.B** was built modifying **5HK1–Ph.A** in the Discovery Studio interface using the available pharmacophore edition functionalities, specifically the averaging, the tolerance edition tool, and the feature customization functionality which was used to exclude certain substructures from the amidine and guanidine default mapping definition of PI that did not show basicity following the prediction of both ACD-classic and ACD-Galas (ACD/Labs, 2014). Langer–Ph, developed in Catalyst, now included in the Discovery Studio platform, was reproduced thanks to the definitions, coordinates, tolerances and weights included in its publication (Laggner et al., 2005). Zampieri–Ph and Banister–Ph were reproduced deriving the feature positions that fulfill the published distances and angles and setting a default constrain radius of 1.6Å for the features. In the case of Zampieri–Ph, the angle of the projection point of the HBA feature was not reported, thus no location constrain was set for that projection point to avoid filtering out any hits of the original Zampieri–Ph. Regarding Banister–Ph, although it was built with the Phase program, Catalyst equivalent features were set for the different pharmacophoric points. In the case of the HBA and the Aromatic Ring (AR) features, as no directionality information was described, again the projection points of those features were left without location constrains. Gund–Ph, originally built using the Sybyl package, was reproduced in Discovery Studio using the given coordinate points (Gund et al., 2004). To be as accurate as possible in replicating the original features, the default tail definition of the Catalyst HBA feature was modified, accepting only the mapping to nitrogen atoms. Thus, the new HBA feature could be used to map the nitrogen location and the provided projection point of the hydrogen bond between the nitrogen and the receptor. To solve the issue of two normal vectors defining the AR, and understanding them as an attempt to map a pi-pi stacking from both sites of the ring, two Catalyst pharmacophores were built: one with the projection point on one side and the other with the projection on the other, requiring the fitting of both pharmacophores at the same time. Again a default constrain radius of 1.6 Å was set for all features except for the HBA projection point where the default radius is 2.2 Å. Oberdorf–Ph could not be reproduced, as no distances, angles or feature coordinates were provided by the authors.

### Screening Methods

The generated multiconformational database with 3,707,672 conformers was screened with the Ligand Pharmacophore Mapping protocol launched from the Pipeline Pilot 2016 interface (Dassault Systèmes BIOVIA, 2016b), were each conformation was mapped separately and only the best mapping solution was returned for each of them, keeping finally only the mapped conformation with the best FitValue for each compound. Further, typical virtual screening conditions were used in the calculation: the omission of any feature was not allowed, and both rigid fit between each ligand conformation and the pharmacophore, as well as flexible fit, where slight conformational modifications are allowed to better fit the pharmacophore, were applied. In the case of the Langer–Ph, the published affinity prediction conditions were also used for screening, using the published weights and setting in this case the maximum number of omitted features to any. In the case of Gund–Ph to achieve the double directionality of the aromatic feature, we screened compounds first with a pharmacophore having the AR pointing to the direction of Tyr103, as determined after the pharamacophore-receptor alignment: Gund-up–Ph, and then we filtered the resulting conformations in place and without fitting, with a second pharmacophore equal to Gund-up–Ph but with the inverted projection point of AR.

For the docking studies, the LibDock program (Diller and Merz, 2001; Rao et al., 2007) implemented in Discovery Studio 16 was used, taking the prepared subunit B of the 5HK1 structure and the generated multi-conformational database of ionized compounds. A Site Sphere of 10 Å centered on the crystallized PD144418 ligand was defined and the docking grid was calculated using 1000 hotspots. No minimum cut-off value was set for the LibDockScore and up to 100 ligand poses could be saved for each ligand, but a filter requiring a charge interaction of the output poses with Glu172 was established to lower the number of possible solutions, as this interaction is the strongest interaction found in the crystallized structure (Schmidt et al., 2016) and mutation of Glu172 has been proven to abolish binding (Seth et al., 2001). Additionally, to ensure a proper orientation of the ligand, a hydrogen bond as part of the electrostatic salt-bridge interaction was also required (Bissantz et al., 2010). Finally poses with unfavorable interactions were filtered out. The remaining LibDock settings were left to their default values, and to score the resulting poses, the following seven scoring functions as implemented in Discovery Studio 16 were used: LigScore1, LigScore2 (Krammer et al., 2005), PLP1, PLP2 (Gehlhaar et al., 1995), Jain (Jain, 1996), PMF (Muegge and Martin, 1999), and PMF04 (Muegge, 2006).

### Human Sigma-1 Receptor Radioligand Assay

The binding properties of the 25,676 compounds to human σ_1_R were studied in transfected HEK-293 membranes using [^3^H](+)-pentazocine (Perkin Elmer, NET-1056) as the radioligand. The assay was carried out with 7 μg of membrane suspension, [^3^H]-(+)-pentazocine (5 nM) in either absence or presence of either buffer or 10 μM haloperidol for total and non-specific binding, respectively. Binding buffer contained Tris-HCl (50 mM, at pH 8). Plates were incubated at 37°C for 120 min. After the incubation period, the reaction mix was transferred to MultiScreen HTS, FC plates (Millipore), filtered and plates were washed (3 times) with ice-cold Tris–HCl (10 mM, pH 7.4). Filters were dried and counted at approximately 40% efficiency in a MicroBeta scintillation counter (Perkin-Elmer) using EcoScint liquid scintillation cocktail. The distribution of activities obtained is indicated in Table 1.

**Table 1:** Experimentally determined σ1R affinity range distribution of compounds in the dataset of 25,676 unique structures used for virtual screening and validation of the different models.

σ1R affinity range, Ki (nM)	#compounds
<50	1620
50–100	707
100–150	430
150–200	298
200–250	235
250–300	165
300–350	110
350–400	114
400–450	127
450–500	99
500–550	91
550–600	96
600–650	93
650–700	120
700–750	135
750–800	107
800–850	84
850–900	50
900–950	54
950–1000	31
>1000	20,910


### Evaluation of Screening Performance

For evaluating the effectiveness of the different models, well-known metrics were used. The Enrichment Factor (*EF^x%^*) measures the density of active compounds that can be found at a given fraction of the model-ordered database in comparison to a random selection. It is calculated by Equation (1), where *Actives*^x%^_Selected_ is the number of active compounds found at top x% of the database screened, following the model ranking; *N*^x%^_Selected_ is the number of compounds at top x% of the database; *Actives*_Total_ is the number of active ligands in entire database; and *N*_total_ is the number of compounds in the entire database. A major drawback of the Enrichment Factor, that turns it unsuitable for comparison of screening performance among different databases, is its dependency on the ratio between active and inactive compounds. However, it allows a ranking of different models for the same database (Truchon and Bayly, 2007).

$$EF^{x\%}=\frac{Actives_{Selected}^{x\%}/N_{Selected}^{x\%}}{Actives_{Total}/N_{total}}\;\;\;\;\;\;\;\;\;\;\;\left(1\right)$$

The Hit Rate (HR^x%^) corresponds to the ratio of known hits found within the top x% and it is defined as the quotient of the real EF and the ideal EF (Hamza et al., 2012).

Sensitivity (TPR) is the fraction of correctly identified active compounds within the selected top x%.

Specificity (TNR) is the fraction of correctly identified inactive compounds within that x%.

The Receiver Operator Characteristic (ROC) curve plots sensitivity (true positive rate) versus specificity at all possible selection thresholds (Fawcett, 2006). The area under its curve (ROC-AUC) is a practical and objective way of measuring the performance of screening models, being independent of the balance of active and inactive compounds present in the database. ROC-AUC values range from 0.0 to 1.0, with 0.5 meaning random selection.

### Similarity Calculations

Extended-Connectivity Fingerprints (Rogers and Hahn, 2010), Functional-Class Fingerprints and MDL public keys (Durant et al., 2002) as implemented in Pipeline Pilot were used as structural descriptors. All pairwise Tanimoto distances among compounds of each set were calculated and statistical values and histogram frequencies were obtained with implemented protocols.

## Results

**FIGURE 2:**
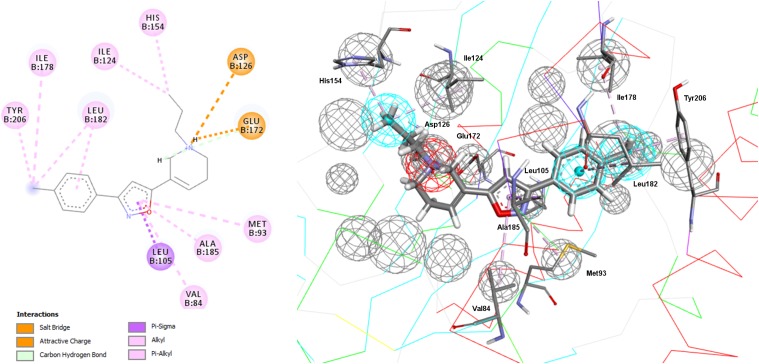
New pharmacophore model **5HK1–Ph.A** based on receptor-ligand interactions. The model consists in one PI feature (red), three HYD features (blue), and twenty-one Excluded Volumes (gray).

As a first step, a new σ1R pharmacophore model based on the receptor-ligand interactions observed in the 5HK1 crystal structure was automatically built. Only four out of the ten pharmacophoric features present in PD144418 were chosen by the algorithm as being the most characteristic and selective ones. Those were one PI feature and three HYD features, two on one side of the PI with distances from 7 to 13 Å and one on the other side at 3.7 Å ± 0.8 Å. The PI feature stands for the ionic interaction between the amine of PD144418 and Glu172 and Asp126; the HYD on one side for the hydrophobic interaction of the propyl chain with Ile124 and His154; and the two other HYD features for the interactions of the phenyl ring and the methyl with Leu182, Tyr206, and Ile178. These features together with the excluded volumes constituted the new σ1R pharmacophore model **5HK1–Ph.A** (Figure 2).

Comparing **5HK1–Ph.A** with previously described models, we found that it perfectly matched the distances of Glennon–Ph. Langer–Ph just differed by having one additional HYD feature, while distances and angles were almost in perfect overlap with the new model (an RMS displacement of 1.1 Å if disregarding the additional HYD1 feature, Figure 1). This supports the feasibility of building ligand-based global models that account for receptor interactions, as well as HypoGen’s model building power when a proper diverse training set with a wide activity range is selected. The fact that the additional HYD feature present in Langer–Ph (HYD1) was not necessary for σ1R binding, and could be replaced by other non-hydrophobic chemical groups, had already been observed for some of our σ1R ligand families. For example, in a series of 4-aminotriazole derivatives (Díaz et al., 2015), the HYD1 feature was reported not to be covered by high affinity ligands; instead, triazole nitrogen atoms were present in that region.

**FIGURE 3:**
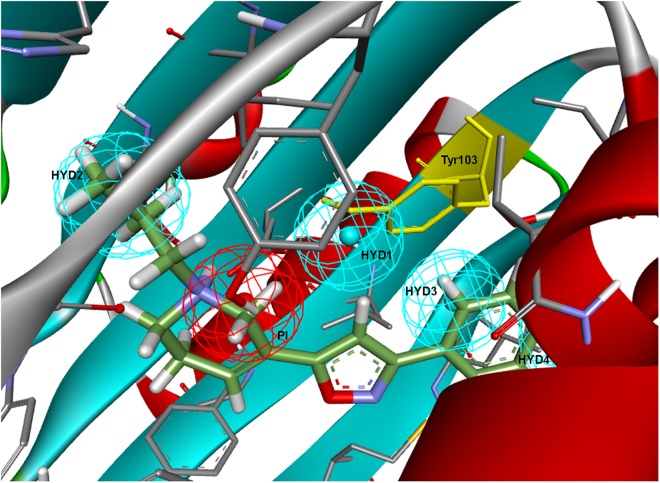
Langer–Ph positioned in the active site of the σ1R. Note that HYD1 collapses with Tyr103 (in yellow).

To further determine whether HYD1 and its position may be dispensable (although it can account for the interaction of particular compound families), Langer–Ph was displaced and positioned into the σ1R active site in two different ways. As a first option the within Discovery Studio available pharmacophore alignment algorithm was used. The second strategy entailed a rigid fitting of PD144418 into the pharmacophore, allowing the omission of one feature, followed by the displacement of the fitted structure to its crystallographic position, displacing at the same time the pharmacophore itself. In both cases, HYD1 turned out to be located directly over Tyr103. This implies that the conformation of a ligand that fulfills the geometrical disposition of the five features that make up the Langer–Ph would be positioned in a way that would at least initially clash with the crystallized σ1R (Figure 3).

**FIGURE 4:**
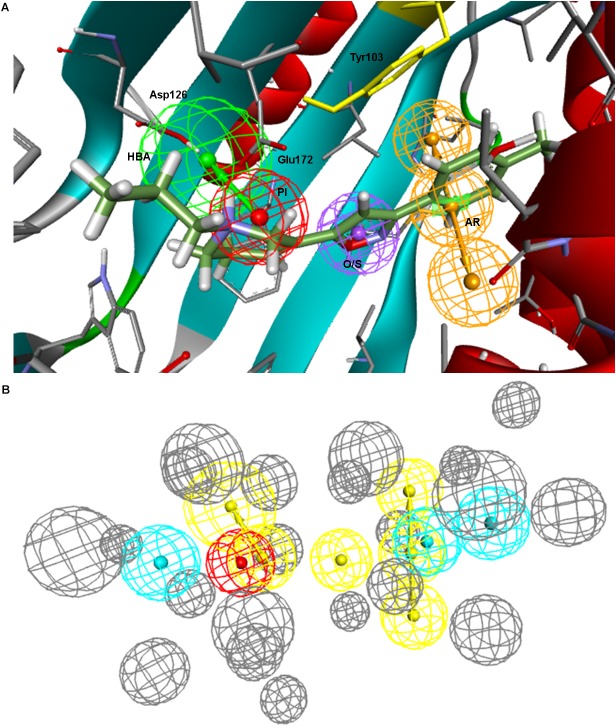
**(A)** Gund–Ph positioned in the active site of the s1R by rigid ligand alignment. The AR features capture the pi-stacking with Tyr103, but there is no aromatic side chain on the other side for the interaction on the opposite direction; **(B)** Gund–Ph (yellow) overlapped with **5HK1–Ph.A.**

Going over to the remaining pharmacophore models that have in common the presence of an additional polar feature, Gund–Ph differed mainly by the absence of a HYD feature next to the PI and by the so-called secondary binding region defined by the presence of an oxygen or sulfur atom. After a rigid fit of Gund–Ph to the crystallized PD144418 model, we found that the AR did coincide with one of the HYD features of **5HK1–Ph.A** (Figure 4). Looking at the receptor, we observed that Tyr103 was actually pi-stacking with the phenyl ring of the ligand, thus the aromatic feature in this position captured a ligand-receptor interaction, although only in one direction, since there was no other aromatic ring facing the phenyl from the other site. As for the directionality of the hydrogen bond established by the nitrogen, it reflected the interaction of the basic amine that may receive a hydrogen atom from either Glu172 or Asp126. In comparison to Langer–Ph, there was no HYD feature on the other site of the PI. Finally, the polar feature, defined in this case by the presence of an oxygen or sulfur atom, can be found in ligands such as PD144418, which was among those used to derive the pharmacophore, but it did not reflect a binding interaction, as the oxygen of the isoxazole ring does not show any polar interaction with the receptor.

**FIGURE 5:**
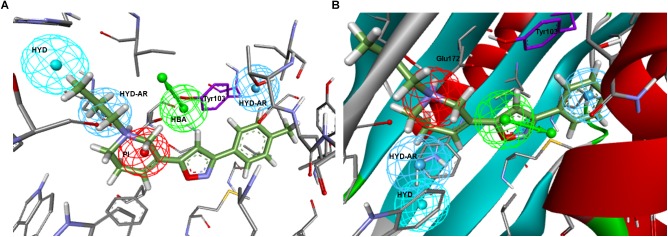
**(A)** Zampieri–Ph place in the σ1R binding site resulting from alignment with **5HK1–Ph.A.** HBA collapses partially with Tyr103 and the crystallized PD144418 does not fulfill more than 2 features; **(B)** Placement resulting from the rigid fit of PD144418 to Zampieri–Ph and displacement of the whole set to the crystallographic position of the ligand. Space constrains can be observed for the non-fitted HYD and HYD-AR features.

Regarding Zampieri–Ph, no more than two features could be aligned simultaneously to **5HK1–Ph.A** when using the pharmacophore alignment algorithm. Only one of the solutions remained within the binding site region delimited by the exclusion volumes after the alignment, but in this case the location of the HBA would partially collapse with Tyr103 and the crystallized PD144418 would not fulfill more than two features in that disposition (Figure 5). On the other hand, a rigid fit of PD144418 was only achieved allowing the omission of two features, and when displacing the solution that mapped the PI feature to the crystallographic position of the ligand, space constrains could be observed for the non-fitted HYD and HYD-AR features of the pharmacophore.

**FIGURE 6:**
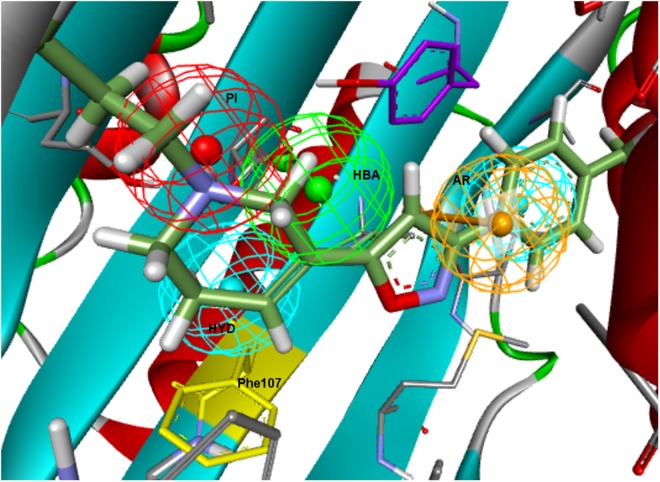
Banister–Ph positioned in the binding site by rigid ligand-fit and displacement. The HYD under the PI might stand for interactions with other HYD aminoacids, mainly with Phe107 (in yellow).

Finally, all Banister–Ph features were mapped by PD144418 except for the HBA, although with a considerable low FitValue (Figure 6). An HBA in the specified position might represent a second polar interaction with Glu172, but this interaction was particular to the chemistry used to derive the model and does not seem to be always required for binding. The HYD feature next to the PI having an equivalent location to **5HK1–Ph.A** or to Langer–Ph was missing, but instead a second HYD that might stand for interactions with other hydrophobic aminoacids (Phe107) was found.

**FIGURE 7:**
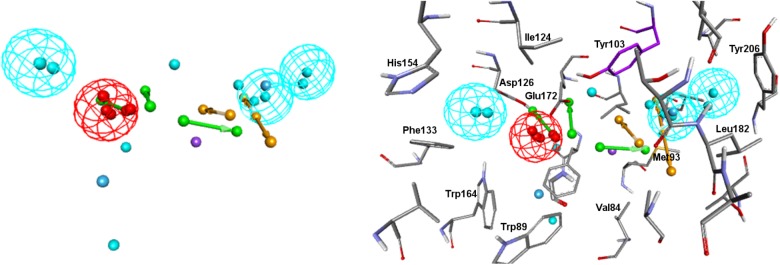
Visualization of the five pharmacophore models (Langer, Gund, Zampieri, Banister and 5HK1-Ph.A) overlapped in the binding site. Location spheres are only shown for **5HK1–Ph.A**.

Visualizing the five pharmacophore models overlapped in the σ1R binding site (Figure 7), we can conclude that they all have identified the important ionic interaction (PI), and coincide in placing a HYD or HYD aromatic site that has turned out to be the space defined by residues Tyr103, Leu105, Leu95, Tyr206, Leu182, and Ala185 and delimited by helices α4 and α5. More ambiguity was observed in the location of the other HYD region, which is not defined in Gund–Ph and has different placements in Banister’s and Zampieri’s models. Only Langer–Ph and the new structure-derived **5HK1–Ph.A** place it at the bottom of the β-barrel, near Asp126. Regarding the polar feature present in three of the models, it might likely reflect regions where a polar group can be tolerated rather than necessary interactions for binding.

In order to experimentally validate and test the performance of the different models, a 3D multiconformational database of 25,676 unique structures was built. They belong to ESTEVE’s internal compound library and have been characterized over the years for σ1R binding (displacement of [3H]-(+)-pentazocine in HEK-293 membranes transfected with human σ1R (DeHaven-Hudkins et al., 1992)). The compound dataset comprises compounds within all the affinity ranges, as indicated in Table 1. It is worth noting that almost half of the compounds considered active for the σ1R (Ki < 1 μM) are high affinity compounds with Ki < 100 nM.

**FIGURE 8:**
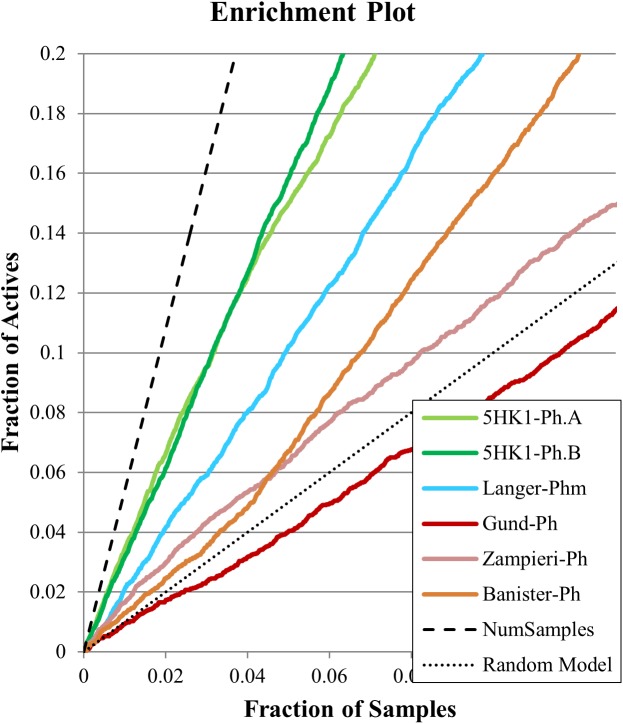
Enrichment plots for the six evaluated pharmacophore models for the first 10% of selected samples.

The resulting multiconformational database (3,707,672 conformers) was screened with the five pharmacophore models applying both rigid and flexible fit. In the case of Langer–Ph, affinity prediction conditions were also tested. In the case of Gund–Ph two options were considered: compounds fulfilling just the directionality of the aromatic feature pointing to Tyr103 (Gund-up–Ph) and compounds with an aromatic feature accessible from both sites, which corresponds to the original definition (Gund–Ph). We then calculated for all the models the sensitivity, specificity, enrichment values and hit rates at 1, 5, and 10% of the database, and the area under the ROC curve (ROC-AUC). Results are displayed in Table 2 and Figure 8. Gund’s and Zampieri’s models failed to discriminate actives from inactives, having ROC-AUC values scarcely above 0.5. Both Gund–Ph and Gund-up–Ph were equally unsatisfactory, probably due to the model simplicity, since both active and inactive compounds were equally able to fit the pharmacophore (high sensitivity and low specificity values), both with similar FitValues translating in enrichment factors around 1. Zampieri–Ph, on the contrary, had a low true positive rate, suggesting that the hypothesized features in the specified arrangement were not fulfilled by a high percentage of σ1R binders. The very low enrichment factors tending to 1 already at the 10% of the ranked compounds indicates that inactive compounds suited the model almost as well as active ones. Both facts, together with the difficulties in the pharmacophore-receptor alignment, may indicate that the lack of success shown by ROC-AUC values was due to a feature disposition that does not geometrically map the key σ1R-ligand interactions.

**Table 2:** Area under the ROC curve, sensitivity, specificity, enrichment factors and hit rates at 1%, 5% and 10% of screened compounds using six different pharmacophore models, with both rigid and flexible fit.

	ROC AUC	Sensitivity (TPR)	Specificity (TNR)	EF^1%^	EF^5%^	EF^10%^	HR^1%^	HR^5%^	HR^10%^
**5HK1–Ph.A**	0.65	0.45	0.84	3.44	3.00	2.66	63.8	55.6	49.3
**5HK1–Ph.A flex.**	0.66	0.5	0.80	3.40	2.79	2.43	63.1	51.7	45.1
**Langer–Ph**	0.67	0.53	0.80	2.10	2.04	2.04	38.9	37.8	37.8
**Langer–Ph flex.**	0.71	0.65	0.76	2.41	2.15	2.10	44.7	39.9	38.9
**Langer–Ph AffPred.^a^**	0.73			1.97	1.93	1.91	36.5	35.8	35.4
**Gund–Ph**	0.52	0.71	0.34	0.94	0.81	0.85	17.5	14.9	15.8
**Gund–Ph flex.**	0.51	0.74	0.33	0.90	0.99	0.88	16.7	18.4	16.3
**Gund-up–Ph**	0.52	0.78	0.31	0.92	0.88	0.88	17.1	16.3	16.3
**Gund-up–Ph flex.**	0.52	0.8	0.3	0.82	0.84	0.91	15.2	15.6	16.9
**Zampieri–Ph**	0.51	0.16	0.87	1.66	1.28	1.18	30.8	23.7	21.9
**Zampieri–Ph flex.**	0.52	0.21	0.83	1.68	1.33	1.15	31.2	24.7	21.3
**Banister–Ph**	0.71	0.79	0.63	1.30	1.34	1.60	24.1	24.9	29.7
**Banister–Ph flex.**	0.76	0.82	0.61	1.30	1.16	1.35	24.1	21.5	25.0
**5HK1–Ph.B**	0.85	0.94	0.63	3.17	3.17	3.10	58.8	58.8	57.5
**5HK1–Ph.B flex.**	0.83	0.95	0.59	2.43	2.67	2.56	45.1	49.5	47.5


*^a^In the case of Langer–Ph affinity prediction conditions have also been tested.*

On the other hand, Langer–Ph, Banister–Ph and the new **5HK1–Ph.A** behaved approximately equal in discriminating active versus inactive compounds, either by applying rigid or flexible fit, with an almost equal poor to fair accuracy based on ROC-AUC values around 0.7. They differed, however, in their sensitivity to specificity ratio. Banister–Ph had a high sensitivity, being able to recover around 80% of the hits, but at the cost of selecting many false positive compounds. Although the final area under the ROC curve was quite fair, enrichment factors up to 10% of the ranked compounds were barely above one. Accordingly, the presence of the features in the reported positions with a tolerance radius of 1.6 Å seems to be common to active compounds and fair enough to distinguish them from inactives, but the predictability is low when considering only compounds with the best adjustments to reported distances and angles. Oppositely, Langer–Ph and **5HK1–Ph.A** managed high specificity values, with lower though acceptable true positive rates and enrichment factors between 2 and 3. Thus, both models were able to differentiate between actives and inactives, both globally and considering only best fitting compounds. In fact, **5HK1–Ph.A** surpassed Langer–Ph in enrichment and hit rate values, with an average hit rate above fifty percent up to a 10% of ranked compounds, meaning that five to six out of each 10 compounds selected by the model show affinity for the σ1R. In general, flexible fits seemed to perform slightly better in terms of ROC-AUC but not when looking at enrichment factors. This small difference may be due to the higher number of compounds fitting the model thanks to this flexibility, conferring some advantage over random at higher fractions of selected compounds. Finally, Langer–Ph under affinity prediction conditions showed comparable results to Langer–Ph using a flexible fit.

**FIGURE 9:**
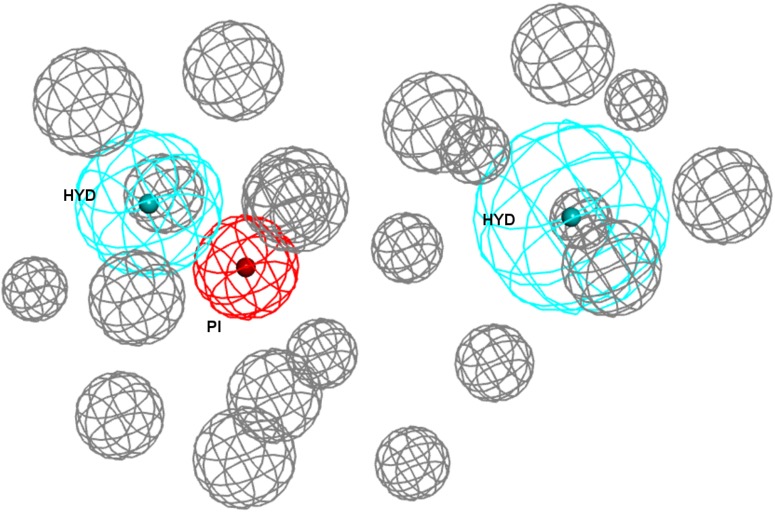
**5HK1–Ph.B** pharmacophore.

Taking into consideration the binding site region (mainly built by amino acids exerting apolar interactions with the ligand) and receptor-ligand interactions automatically retrieved in the **5HK1–Ph.A**, we suspected that the two contiguous HYD features could be due to the nature of the ligand complexed in the crystal structure rather than to a real requisite for σ1R binding. Therefore we decided to modify **5HK1–Ph.A** in order to average the two mentioned HYD features into a new one, placed at their center. This was done by increasing the tolerance to 3 Å to allow the fitting of any compound amenable to HYD interactions at that region, but without exceeding the surface delimited by the excluded volumes. Further, the tolerance of the HYD feature at the other site of the PI group was increased to 2.2 Å, which approximately corresponds to the available receptor cavity, and excluded volumes were left the same. Additionally the PI feature was customized to exclude certain substructures from the amidine and guanidine default PI definition. With all these parameters a new pharmacophore, **5HK1–Ph.B**, was generated (Figure 9) and used to screen the same 3D multiconformational database applying again both rigid and flexible fit. The new results and statistical measures can be found as well in Table 2 and Figure 8. We found that by merging the two HYD features sensitivity increased to optimal values (around 0.95), which means that **5HK1–Ph.B** is able to recognize almost all binders and without a substantial decrease, neither in precision nor in specificity, in comparison to the previous models. The higher sensitivity translated into a ROC-AUC value above 0.8, indicating a good statistical accuracy. Rigid fit surpassed flexible fit. Further enrichment factors and hit rates of the new models at screening percentages below 10% of the database are quite comparable to the best ones obtained previously. This leaves **5HK1–Ph.B** as the best σ1R pharmacophore model among those assayed in this study in light of our internal, experimental *in vitro* data.

**FIGURE 10:**
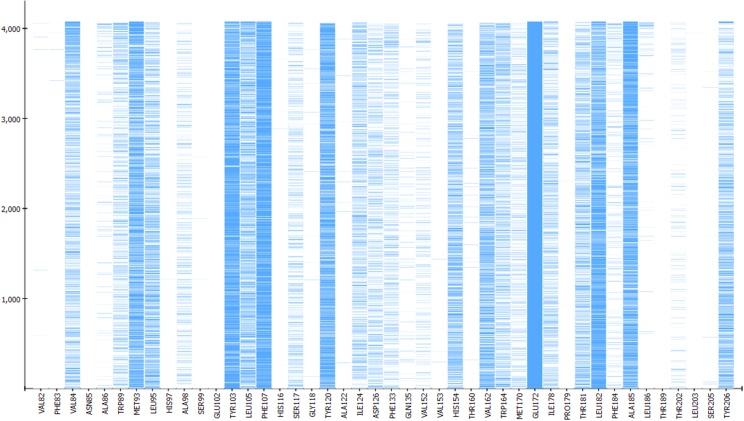
Aminoacids exerting favorable interactions with σ1R ligands with the best pose scored by –PMF04. Together with Glu172; Met93, Tyr103, Phe107, Tyr120, Leu182, and Ala185 are important for ligand recognition.

In addition to the pharmacophore models it was deemed interesting to perform a docking-based virtual screening using the coordinates of the crystal σ1R structure. For that purpose the 25,676 compounds were ionized for pH values greater than 5 to generate a new conformational database with 7,573,004 conformers that were docked using LibDock (Diller and Merz, 2001; Rao et al., 2007) as described in the experimental section. As shown in Table 3, the docking process was able to differentiate active from inactive compounds with fair ROC-AUC values around 0.77 for the different scoring functions, providing better sensitivity than specificity. That is, it generated more false positives than false negatives. The main difference among the scoring functions was found in enrichment values in the first 10% of ranked compounds, where -PMF04, LigScore2_Dreiding and Jain achieved the higher values. With the best scored pose of σ1R ligands (obtained with -PMF04), receptor-ligand interaction analysis was performed (Figure 10). It can be appreciated that, together with Glu172, other aminoacids such as Met93, Tyr103, Phe107, Tyr120, Leu182, and Ala185 are important for ligand recognition.

**Table 3:** Area under the ROC curve, sensitivity, specificity, enrichment factors and hit rates at 1, 5, and 10% of ranked compounds after docking and scoring by seven different scoring functions.

	ROC-AUC	EF^1%^	EF^5%^	EF^10%^	HR^1%^	HR^5%^	HR^10%^
-PLP1	0.77	1.74	1.99	2.18	32.3	36.9	40.3
-PLP2	0.77	1.72	2.1	2.14	31.9	38.9	39.7
-PMF	0.76	1.3	1.72	1.9	24.1	31.9	35.2
-PMF04	0.77	2.81	2.34	2.27	52.1	43.4	42.1
Jain	0.78	1.87	2.3	2.4	34.7	42.7	44.5
LigScore1_Dreiding	0.74	1.51	1.6	1.82	28	29.7	33.8
LigScore2_Dreiding	0.75	2.62	2.36	2.2	48.6	43.8	40.8


*–PMF04 shows the best results throughout the different indicators. Sensitivity (TPR): 0.85 Specificity (TNR): 0.59.*

When comparing pharmacophore-based and docking-base screenings, pharmacophore search using **5HK1–Ph.B** outperformed docking results in all evaluated parameters. **5HK1–Ph.A** also showed a better performance than docking when looking at enrichment values, although with an opposite sensitivity-specificity profile. This may be due to the rigidity of the crystal structure in the docking process, as opposed to the feature tolerances in the pharmacophore model. Additionally, the importance of the HYD interactions characteristic of the σ1R and the penalty for desolvating ligands with polar atoms may be not well captured by the tested scoring functions, whereas the pharmacophore model directly requires HYD groups to fill those regions up.

**FIGURE 11:**
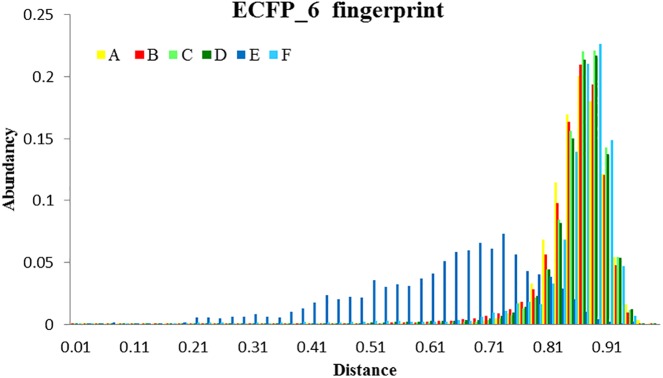
Analysis of the diversity of the compounds with σ1R affinity compared to the diversity of the whole database and compared as well with the diversity shown by the analogs of a lead compound. The diversity obtained by the pharmacophore selection (C) is comparable to that of the whole database (A). A, All library compounds; B, Active compounds in the library; C, first 10% of selected compounds by the **5HK1–Ph.B** model; D, True positives within this 10%; E, 88 analogs of S1RA (E52862); F, First 88 ranked compounds by the **5HK1–Ph.B** model.

Finally, to assess the value of the **5HK1–Ph.B** model not only in terms of effectiveness but also in its potential to capture diversity, we calculated all pairwise Tanimoto similarities for different subgroups of compounds, as depicted in Table 4 and Figure 11. Three structural descriptors were used: Extended-Connectivity Fingerprints and Functional-Class Fingerprints with diameters four and six (that is maximal distance in bond length considered for the generation of the atom-centered substructural features encoded), and the MDL public keys implemented in Pipeline Pilot. Out of those pairwise distances, the average, median and mode distance values were also determined. Four subgroups were devised: (i) all the 25,676 compounds in the library; (ii) all the active compounds; (iii) the first 10% of selected compounds by the **5HK1–Ph.B** model; and (iv) the true active compounds within this 10%. As reference values for a selection of analogs we considered 88 active analogs of the σ1R antagonist in clinical development (S1RA; E52862) (Díaz et al., 2012) as well as the first 88 ranked compounds by the **5HK1–Ph.B** model. We first found that calculated distances of both Extended-Connectivity and Functional-Class fingerprints with diameter six exhibited slightly greater distances than those calculated with diameter four, and both of them returned higher values than those determined using MDL Public Keys. Interestingly, however, the same conclusions can be drawn with all of them: active compounds among the library are very diverse, with average, median and mode distances quite close to those exhibited by the whole library, which confirms the structural variety of σ1R binders. The same degree of diversity was also observed for the first 10% compounds selected by the **5HK1–Ph.B** model, considering actives and inactives or only active compounds among the selected. In fact, statistical values obtained for the true positives among this 10% were almost equal to the values obtained for all the actives in the library. It is remarkable that the first 88 active compounds ranked by the model were able to reach high average distances, whereas the 88 analogs of S1RA showed clearly lower values. This reinforces the aforementioned ability of **5HK1–Ph.B** to discriminate binders even when there are high structural differences among them.

**Table 4:** Average (μ), median (Me), and mode (Mo) pairwise Tanimoto distance values for five different subgroups: 88 analogs of the lead compound S1RA (E52862); the first 10% of selected compounds by the 5HK1–Ph.B model; the true active compounds within this 10%; all database compounds; all active compounds in the database.

	ECFP_6			ECFP_4			FCFP_6			FCFP_4			MDLPublicKeys		
	μ	M_e_	M_o_	μ	M_e_	M_o_	μ	M_e_	M_o_	μ	M_e_	M_o_	μ	M_e_	M_o_
S1RA Analogs (88)	0.63	0.66	0.67	0.55	0.56	0.50	0.57	0.59	0.50	0.45	0.45	0.50	0.18	0.17	0.11
First 88 actives	0.85	0.87	0.89	0.82	0.84	0.86	0.82	0.85	0.86	0.76	0.78	0.75	0.42	0.44	0.50
First 10%	0.87	0.87	0.88	0.83	0.84	0.83	0.84	0.85	0.83	0.77	0.78	0.80	0.45	0.46	0.50
TP in the 10%	0.86	0.87	0.88	0.83	0.84	0.83	0.83	0.84	0.83	0.76	0.77	0.75	0.45	0.47	0.50
All database	0.89	0.89	0.89	0.86	0.87	0.86	0.86	0.87	0.86	0.82	0.82	0.80	0.52	0.52	0.50
All actives	0.86	0.87	0.88	0.82	0.84	0.83	0.83	0.84	0.83	0.76	0.77	0.75	0.45	0.46	0.50


## Discussion

After the publication of the σ1R crystal structure, a new avenue was open for the derivation of accurate models, either by generating new receptor-ligand derived pharmacophore models or by using it for docking studies. In order to show how this information could help in the design of new σ1R ligands we decided to use it for the generation of new pharmacophoric models of general applicability. Two models were developed: The first one, **5HK1–Ph.A,** was obtained by an algorithm that identifies the most important receptor-ligand interactions including as well excluded volumes based on atom location on the protein. The second, **5HK1–Ph.B,** resulted from a manual edition of the first one mainly by merging two HYD features that we thought match the particular structure of one co-crystallized ligand more than specific requirements of the binding site.

In order to compare these new models with the information provided by previously published σ1R pharmacophore models (Langer–Ph, Gund–Ph, Zampieri–Ph, Banister–Ph), we carried out a study involving a set of 25,676 structures of our internal database that had been experimentally screened for σ1R affinity in a binding assay of [^3^H]-(+)-pentazocine displacement and displayed a wide range of activities and structural diversity.

All the pharmacophoric models assessed identified the important ionic interaction (PI) of ligands with Glu172 and placed a HYD or HYD aromatic site in the same region that turned out to be the space defined by residues Tyr103, Leu105, Leu95, Tyr206, Leu182, and Ala185 and delimited by helices α4 and α5. More ambiguity was observed in the location of the other HYD region, which is not defined in Gund–Ph and has different placements in Banister’s and Zampieri’s models. Only Langer–Ph and the new structure-derived **5HK1–Ph.A** and **5HK1–Ph.B** place it at the bottom of the β-barrel, near Asp126.

Finally, we also docked the ionized database using a high throughput docking technique and scored the resulting poses with seven different scoring functions. With the best scored pose of σ1R ligands obtained with the best scoring function (-PMF04), receptor-ligand interaction analysis was performed and it was determined that, together with Glu172, other aminoacids such as Met93, Tyr103, Phe107, Tyr120, Leu182, and Ala185 are important for ligand recognition.

Statistical performance measures were obtained with all the models generated, including Hit Rate (ratio of known hits found within the top x%), sensitivity (fraction of correctly identified active compounds), specificity (fraction of correctly identified inactive compounds) and the area under the Receiver Operator Characteristic Curve (ROC-AUC, which plots the true positive rate against the false positive rate at descending model’s scores). When comparing all these parameters throughout the different models, **5HK1–Ph.B** emerged as the best model to discriminate between active and inactive compounds, with a ROC-AUC value above 0.8 and enrichment values above 3 at different fractions of screened samples. This means that **5HK1–Ph.B** could be used with the highest confidence in relation to any of the previously available models either in the design of new σ1R ligands or in the virtual screening of large compound collections, where an increased hit-rate ratio is expected.

When comparing pharmacophore-based with docking-based screening, the receptor derived pharmacophore **5HK1–Ph.B** showed better results than the direct docking to the receptor. The superior performance of the pharmacophore screening is not absolutely unexpected as it has already been reported for other targets (Chen et al., 2009) and could be explained by the rigidity of the crystal structure in the docking process, that could be implicitly compensated by the feature tolerances in the pharmacophore model. Additionally, HYD interactions are very relevant in the σ1R binding region and the penalty for desolvating ligands with polar atoms could be not well captured by the docking scoring functions. On the contrary, the pharmacophore model directly requires HYD groups to fill up those regions of the binding site.

It is important to note that σ1R binds a remarkable variety of small molecules with high affinity (<100 nM), as already shown in the literature (Almansa and Vela, 2014). The results reported here were obtained using an internal database of drug-like as well as CNS-oriented molecules with experimentally determined affinities using a homogenous procedure, both for active and inactive compounds. Many of them were generated in the context of Medicinal Chemistry σ1R programs and hence the database contains many diverse scaffolds where small modifications within congeneric series may abolish activity. This situation is not frequently encountered since models are usually generated or validated based on one or a few chemical families active on the target, in front of assumed inactives or decoys obtained by diversity selection of drug-like compounds (Réau et al., 2018). Altogether, the use of a large and diverse compound collection together with accurate structural information provides a sound basis for the generation and validation of predictive models to design new molecules.

While writing this manuscript, a 3D-QSAR model for a pooled dataset of known σ1R antagonists from five structurally diverse chemical families, with 147 compounds for model development and 33 compounds for model validation, has been published (Peng et al., 2018). Interestingly, the X-ray crystal structure of the human σ1R in complex with PD144418 was used to derive the pharmacophore model needed for the structural alignment of the compounds. With this alignment procedure, a predictive 3D-QSAR model for σ1R antagonists was obtained and further validated by virtually screening the DrugBank database of FDA approved drugs. Two approved drugs with high and previously unknown σ1R affinities were identified (diphenhydramine and phenyltoloxamine; Ki = 58 and 160 nM, respectively). Despite the constrained applicability domain of 3D-QSAR to the range of binding affinities and chemical space of the training set ligands, the publication demonstrates as well the success in the use of the X-ray structure for model development, allowing the identification of new drug leads prior to the resource-demanding tasks of chemical synthesis and experimental biological evaluation.

Finally, it is important to note that classification of σ1R ligands as agonists or antagonists has been often based on their opposing or counteracting effects on biological systems including cell lines, primary cultures and animals (Cobos et al., 2008; Maurice and Su, 2009; Entrena et al., 2016). Little is known in terms of specific structural features or specific receptor conformations when agonists or antagonists are bound. Ligand-mediated conformational changes distinctive for agonist and antagonist ligands were observed when some reference σ1R ligands were assayed in a σ1R fluorescence resonance energy transfer (FRET)-based biosensor (Gómez-Soler et al., 2014). FRET data also support distinctive interactions as some σ1R antagonists stabilize high-molecular-weight oligomers, while certain agonists suppress oligomerization (Mishra et al., 2015). However, the agonist-bound crystallizes similarly to the antagonist-bound σ1R, and the overall conformation of the receptor does not significantly differ, except for a 1.8 Å shift of helix α4 found when compared the (+)-pentazocine-bound relative to the PD 144418-bound structure (Schmidt et al., 2018). Thus, current structural data are insufficient to comment substantively on the impact of identified receptor-ligand interactions on the functional nature of assayed ligands. This will doubtless be an important area for future research. Going further, elucidation of distinct ligand-driven conformations and regulation of homo-/heteromerization states is poised to be an important area for σ1R structural biology. Importantly, the advent of structural data now allows more rational construct design and analysis for computational work.

## Author Contributions

The manuscript was written through contributions of all authors. All authors have given approval to the final version of the manuscript.

## Conflict of Interest Statement

All authors are full-time employees of ESTEVE Pharmaceuticals S.A. The handling Editor declared a past co-authorship with one of the authors JV.

## Abbreviations

2/3D: 2/3-Dimensional
EF: Enrichment Factor
HR: Hit Rate
HYD: Hydrophobic
HBA: Hydrogen Bond Acceptor
PI: Positive Ionizable
AR: Aromatic Ring
HYD-AR: Hydrophobic Aromatic
ROC: Receiver Operator Characteristic
ROC-AUC: Area under the ROC curve
σ1R: Sigma-1-Receptor
TRP: Sensitivity
TNR: Specificity
ECFP: extended connectivity fingerprints
FCFP: functional class fingerprints
